# Cardiac Metabolic Pathways Affected in the Mouse Model of Barth Syndrome

**DOI:** 10.1371/journal.pone.0128561

**Published:** 2015-06-01

**Authors:** Yan Huang, Corey Powers, Satish K. Madala, Kenneth D. Greis, Wendy D. Haffey, Jeffrey A. Towbin, Enkhsaikhan Purevjav, Sabzali Javadov, Arnold W. Strauss, Zaza Khuchua

**Affiliations:** 1 The Heart Institute, Department of Pediatrics, the University of Cincinnati College of Medicine and Cincinnati Children’s Hospital Medical Center, Cincinnati, OH, United States of America; 2 Division of Pulmonary Medicine, Department of Pediatrics, the University of Cincinnati College of Medicine and Cincinnati Children’s Hospital Medical Center, Cincinnati, OH, United States of America; 3 Department of Cancer Biology, UC College of Medicine, University of Cincinnati, Cincinnati, OH, United States of America; 4 Department of Physiology, School of Medicine, University of Puerto Rico, San Juan, 00936–5067, PR; 5 University of Tennessee Health Science Center, Memphis, TN, United States of America; 6 St. Jude Children's Research Hospital, Memphis, TN, United States of America; 7 Le Bonheur Children's Hospital, Memphis, TN, United States of America; Instituto Nacional de Cardiologia I. Ch., MEXICO

## Abstract

Cardiolipin (CL) is a mitochondrial phospholipid essential for electron transport chain (ETC) integrity. CL-deficiency in humans is caused by mutations in the tafazzin (*Taz*) gene and results in a multisystem pediatric disorder, Barth syndrome (BTHS). It has been reported that tafazzin deficiency destabilizes mitochondrial respiratory chain complexes and affects supercomplex assembly. The aim of this study was to investigate the impact of Taz-knockdown on the mitochondrial proteomic landscape and metabolic processes, such as stability of respiratory chain supercomplexes and their interactions with fatty acid oxidation enzymes in cardiac muscle. Proteomic analysis demonstrated reduction of several polypeptides of the mitochondrial respiratory chain, including Rieske and cytochrome c1 subunits of complex III, NADH dehydrogenase alpha subunit 5 of complex I and the catalytic core-forming subunit of F_0_F_1_-ATP synthase. Taz gene knockdown resulted in upregulation of enzymes of folate and amino acid metabolic pathways in heart mitochondria, demonstrating that Taz-deficiency causes substantive metabolic remodeling in cardiac muscle. Mitochondrial respiratory chain supercomplexes are destabilized in CL-depleted mitochondria from Taz knockdown hearts resulting in disruption of the interactions between ETC and the fatty acid oxidation enzymes, very long-chain acyl-CoA dehydrogenase and long-chain 3-hydroxyacyl-CoA dehydrogenase, potentially affecting the metabolic channeling of reducing equivalents between these two metabolic pathways. Mitochondria-bound myoglobin was significantly reduced in Taz-knockdown hearts, potentially disrupting intracellular oxygen delivery to the oxidative phosphorylation system. Our results identify the critical pathways affected by the Taz-deficiency in mitochondria and establish a future framework for development of therapeutic options for BTHS.

## Introduction

Cardiolipin is a unique mitochondrial tetra-acyl phospholipid that has four fatty acyl chains. In most mammalian tissues the acyl-group composition is highly specific being predominantly comprised of 18-carbon unsaturated acyl chains, the vast majority of which are linoleic acid. A tetralinoleoyl cardiolipin (L_4_CL) is a major form of cardiolipin in the mammalian cardiac mitochondria where it constitutes 80–90% of CLs [reviewed in [1, 2]]. L_4_CL-deficiency in humans results in the multisystem pediatric disorder known as Barth syndrome (BTHS) (OMIM: 302060). The causative BTHS gene, tafazzin (*Taz*), is located on the X-chromosome [3]. Taz is an evolutionarily conserved and ubiquitously expressed mitochondrial transacylase that is required for the final step of CL biosynthesis, including remodeling and generation of L_4_CL species. Mutations in the *Taz* gene result in reduction of L_4_CL and accumulation of CL species with unusual acyl-group compositions with higher degrees of saturation [4, 5].

CL is crucial for mitochondrial protein transport and the recycling machinery. Also, it is an integration site of mitochondrial electron transport chain (ETC) supercomplexes and is essential for optimal activities of individual ETC complexes, ATP-synthase, and adenine-nucleotide translocase [6–9]. Moreover, CL is required to maintain proper cristae architecture in mitochondria [10]. L_4_CL-deficient mitochondria form abnormal, circular or honeycomb-like structures, a characteristic feature of defective mitochondria of striated muscles in BTHS patients and Taz-deficient flies and mice. Cardiolipin deficiency disrupts multiple processes and biological pathways such as fatty acid metabolism, mitochondrial fission and fusion, lipid signaling, apoptosis and autophagy [11, 12].

Recent studies have shown that tetracycline-inducible gene knockdown suppresses *Taz* gene expression by > 90% and results in profound L_4_CL deficiency in cardiac and skeletal muscles of B6.Cg-*Gt(ROSA)26Sor* ^*tm1(H1/tetO-RNAi*:*Taz*,*CAG-tetR)Bsf*^/ZkhuJ, or TazKD mice, mimicking a biochemical phenotype of human BTHS [11–13]. Previous studies have demonstrated that Taz inactivation results in two-fold reduction of enzymatic activity of mitochondrial complex III, while has a little or no effect on the enzymatic activities of complexes I, II, IV and V [12, 14]. Taz knockdown and L4CL deficiency result in a complex cardiac phenotype in mice: approximately half of the embryos die *in utero* between E12.5—E14.5 from fetal heart failure, while surviving TazKD pups are born without any signs of disease; however, survivors develop dilated cardiomyopathy with depressed systolic function by 6–8 months of age [11, 13, 15]. TazKD mice demonstrate a deficit in mitochondrial respiratory capacity caused by deficiency in mitochondrial ETC complex III (C-III) [14, 15]. Reduction of L_4_CL in BTHS alters global gene expression, inducing alternative energy producing pathways, conceivably to compensate for diminished energy output of defective mitochondria [12]. However, detailed molecular analysis of L_4_CL-depleted mitochondria that ultimately leads to development of cardiomyopathy has not yet been done.

Herein, we report results of proteomic studies of cardiac mitochondria in TazKD mice that provide new insights into the pathogenesis of BTHS, unveil compensatory mechanisms and provide new markers for diagnosis.

## Materials and Methods

### Animals

Taz-knockdown mice were maintained as described previously [11, 13]. All mice were 3 month old males of C57BL/6 genetic background. Animals were maintained in micro-isolator cages at 25°C under a 14/10 h light/dark cycle with free access to water and food. Taz knockdown was induced at conception with introduction of doxycycline in rodent chow (625 mg/kg) to females before mating. Genotyping was performed by PCR analysis of tail genomic DNA, as described previously [11]. This study was carried out in strict accordance with the recommendations in the Guide for the Care and Use of Laboratory Animals of the National Institutes of Health. Mice were euthanized by overdose of isoflurane followed by cervical dislocation. The protocol was approved by the Institutional Animal Care and Use Committee of the Cincinnati Children’s Research Foundation (Protocol number: 1D03028).

### Isolation of mitochondria

All reagents were purchased from Sigma-Aldrich, unless otherwise noted. Cardiac mitochondria were isolated from freshly excised hearts as described previously [14] and washed 3 times with ice-cold isotonic buffer composed of 0.3 M sucrose, 10 mM HEPES-K (pH 7.4) and 1 mM EDTA. After the final wash, mitochondria were resuspended in 20 μl of isotonic buffer, and protein concentration was determined using the DC protein assay (BioRad). Preparations were aliquoted (1 mg protein per tube), snap-frozen in liquid nitrogen and kept at -80°C until further analysis.

### Two-dimensional difference gel electrophoresis (2D-DIGE)

2D-DIGE analysis including protein labeling, 2D-electrophoreses, gel analysis and identification of proteins of interest were performed by Applied Biomics (Hayward, CA) using established protocols. Briefly, two independent pairs of wild type (WT) and TazKD mitochondrial preparations (4 samples total) were subjected to two separate 2D-DIGE analyses. A mitochondrial suspension equal to 1 mg protein was treated with 150 μl of lysis buffer containing 30 mM Tris-HCl, pH 8.8, 7 M urea, 2 M thiourea and 4% CHAPS. Samples were sonicated at 4°C, followed by shaking at room temperature for 30 min. Samples were centrifuged at 25,000xg for 30 min, supernatants collected and protein concentration measured. Samples (30 μg of protein) were labeled with 1 μl of 0.2 nmole of CyDye, vortexed and incubated on ice in the dark for 30 min. Labeling reactions were terminated with 1 μl of 10 mM lysine.

An equal amount of paired samples of each genotype labeled with Cy3 and Cy5 were dissolved in sample buffer (7 M urea, 2 M thiourea, 4% CHAPS, 20 mg/ml DTT, 1% Pharmalytes). An equal amount of samples from each genotype was labeled with Cy2 and used as an internal standard. Paired labeled samples were mixed together and subjected to isoelectric focusing (first dimension) using the IPGphore 3 system with Immobiline DryStrips (18 cm, pH 3–11 NL) (GE Healthcare) and then to SDS PAGE (second dimension) on the Ettan DALT system, as recommended by the manufacturer. Gels were scanned immediately after SDS PAGE using Typhoon TRIO (GE Healthcare). Images were analyzed by Image QuantTL software and subjected to in-gel analysis and cross-gel analysis using DeCyder 6.5 software (GE Healthcare). Spots of interest were picked by Ettan Spot Picker (GE Healthcare) based on the in-gel analysis and spot-picking design by DeCyder software. Proteins in the gel spots were digested with modified porcine trypsin protease (Trypsin Gold, Promega). The digested tryptic peptides were desalted by Zip-tip C18 (Millipore). Peptides were eluted from the Zip-tip with 0.5 μl of matrix solution (α-cyano-4-hydroxycinnamic acid, 5 mg/ml in 50% acetonitrile, 0.1% trifluoroacetic acid, 25 mM ammonium bicarbonate) and spotted on the MALDI plate.

MALDI-TOF (matrix-assisted laser desorption ionization time of flight;) and TOF/TOF (tandem MS/MS) were performed on a 5800 mass spectrometer (AB Sciex). MALDI-TOF mass spectra were acquired in reflectron positive ion mode, averaging 2000 laser shots per spectrum. TOF/TOF tandem MS fragmentation spectra were acquired for each sample, averaging 2000 laser shots per fragmentation spectrum on each of the 10 most abundant ions present in each sample (excluding trypsin autolytic peptides and other known background ions).

Both the resulting peptide mass and the associated fragmentation spectra were submitted to GPS Explorer version 3.5 equipped with a MASCOT search engine (Matrix Science) to interrogate the database of the National Center for Biotechnology Information non-redundant (NCBInr). Searches were performed without constraining protein molecular weight or isoelectric point, with variable carbamidomethylation of cysteine and oxidation of methionine residues, and with one missed cleavage allowed in the search parameters. Candidates with either protein score C.I.% or Ion C.I.% greater than 95 were considered significant.

### Relative quantitation by Isobaric protein labeling (iTRAQ) and tandem mass spectrometry

**Fig 1:**
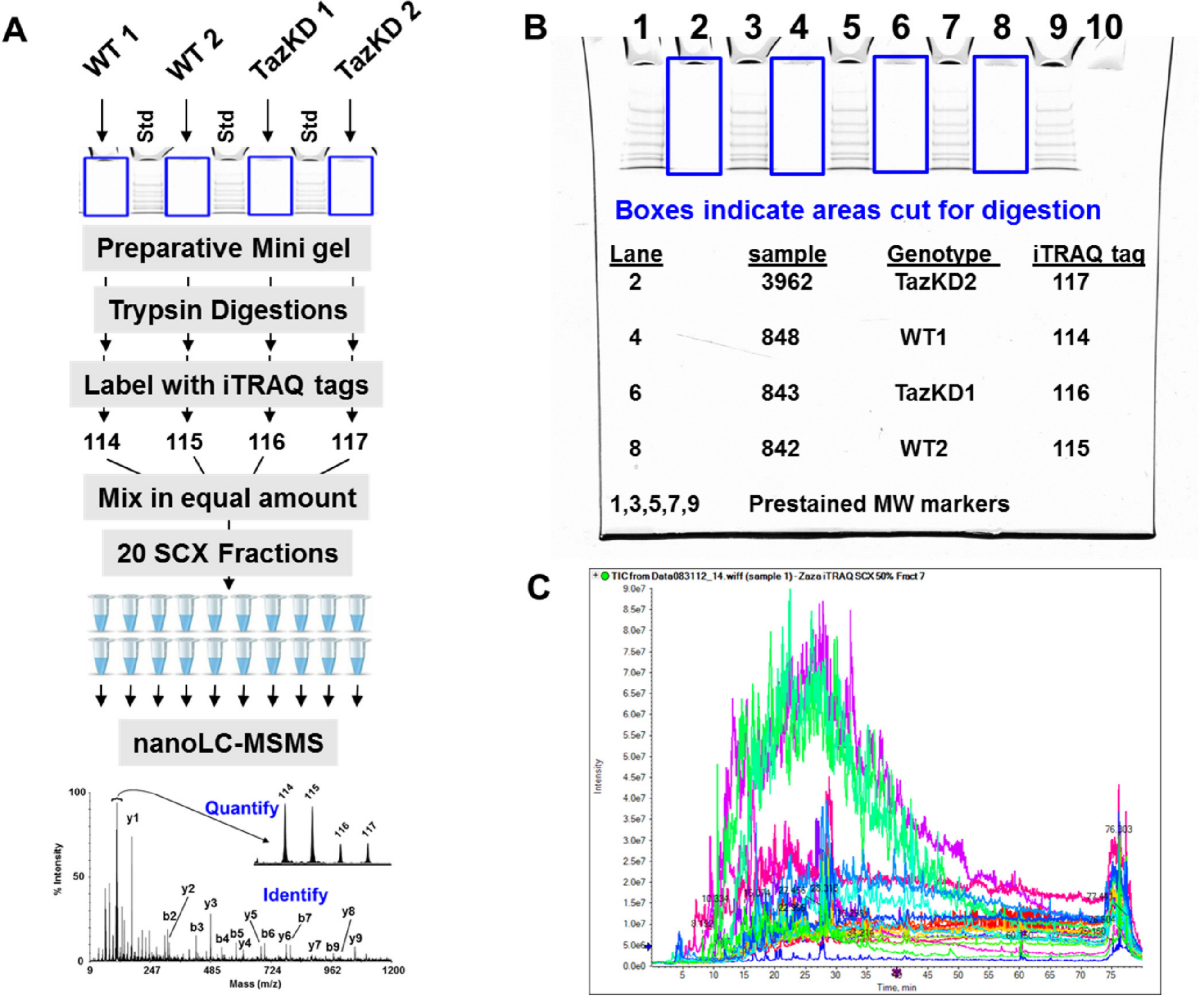
iTRAQ workflow for differential protein profiling in mitochondria from 3-month-old wild type and TazKD mice. (A) Sample preparation workflow from a preparative SDS-PAGE gel, trypsin digestion, iTRAQ labeling, fractionation by strong cation exchange, followed by nanoLC-MS/MS to produce both protein identification by peptide fragmentation data and relative quantitation from the iTRAQ reporter fragment ions. (B) The preparative SDS-PAGE gel with pre-stained molecular weight markers showing the gel regions collected for trypsin digestion. (C) Overlay of the nano-LC-MS total ion current (TIC) from each of the 20 SCX fractions.

Enriched mitochondria from duplicate control and TazKD mouse hearts (4 samples total) were subjected to an isobaric tagging workflow as presented in Fig 1A. The samples were initially solubilized in Laemmli gel buffer with heating to 60°C for 10 min. The protein concentration for each sample was determined using the non-interfering (Ni) protein assay reagents from G-Biosciences (Maryland Heights, MO). Mitochondrial protein samples (50 μg) were loaded onto separated lanes of 4–12% MOPS 1.0 mm mini gel, then electrophoresed for 15 min which was just long enough for the proteins to enter the gel. The gel regions containing the proteins (about 1.5 cm x 2.5 cm) were cut from the gel and subjected to in gel trypsin digestion and subsequent recovery of peptides as described previously [16]. The isolated peptides were tagged using the 4-plex iTRAQ reagents following the vendor (AB Sciex) instructions and as described previously [17]. The 116 and 117 reporter tags were used for the TazKD samples while the 114 and 115 reporter tags were used for the WT control samples. After labeling, the samples were mixed together for subsequent separation and analysis.

Next, 4-plex iTRAQ mixture of peptides was separated into 20 fractions by strong cation exchange (SCX) chromatography on an AKTA purifier HPLC (GE Healthcare, Piscataway, NJ) using a 2.1 x 100 mm PolyLC Polysulfoethyl A column packed with 5 μm beads with 300 Å pores (The Nest Group, Southborough, MA). A 2.0 x 10 mm guard column of the same material was used. The pooled 4-plex iTRAQ labeled peptide mixture was concentrated to 30 μL and re-suspended to a volume to 2 mL with 25 mM ammonium formate (NH_4_HCO_2_), in 25% acetonitrile, pH 2.7. After the pH was adjusted to 2.7 with formic acid, the sample was loaded and washed isocratically for 25 minutes at a flow rate of 0.5 mL/min to remove excess reagent. Retained peptides were eluted with a linear gradient of 0–750 mM ammonium formate (in 25 mM NH_4_HCO_2_ in 25% acetonitrile, pH 2.7) over 60 minutes at 0.5 mL/min. Twenty fractions were collected, frozen and dried in a SpeedVac concentrator. The samples were diluted to 100 μL in 0.1% formic acid, frozen, and dried again. The fractions were each reconstituted in 10 μL in 0.1% formic acid and 5 μL (50%) was evaluated by nLC-ESI-MS/MS.

Nano-electrospray liquid chromatography couple tandem mass spectrometry (nLC-ESI-MS/MS) analyses were performed on a TripleTOF 5600 (ABSciex, Toronto, On, Canada) coupled with an Eksigent nanoLC ultra nanoflow system (Dublin, CA). Fifty percent (5 uL) of each reconstituted SCX fraction was loaded (via an Eksigent nanoLC.as-2 autosampler) onto an IntegraFrit Trap Column (outer diameter of 360 μm, inner diameter of 100, and 25 μm packed bed) from New Objective, Inc. (Woburn, MA) at 2 μl/min in formic acid/H_2_O 0.1/99.9 (v/v) for 10 min to desalt and concentrate the samples. For the chromatographic separation of peptides, the trap-column was switched to align with the analytical column, Acclaim PepMap100 (inner diameter of 75 μm, length of 15 cm, C18 particle sizes of 3 μm and pore sizes of 100 Å) from Dionex-Thermo Fisher Scientific (Sunnyvale, CA). The peptides were eluted using a varying mobile phase (MP) gradient from 95% phase A (FA/H2O 0.1/99.9, v/v) to 40% phase B (FA/ACN 0.1/99.9, v/v) for 70 minutes, from 40% phase B to 85% phase B for 5 minutes and then keeping the same MP-composition for 5 more minutes at 300 nL/min. The nLC effluent was ionized and sprayed into the mass spectrometer using NANOSpray III Source (ABSciex, Toronto, On, Canada). Ion source gas 1 (GS1), ion source gas 2 (GS2) and curtain gas (CUR) were respectively kept at 7, 0 and 25 vendor specified arbitrary units. Interface heater temperature and ion spray voltage was kept at 150 C, and at 2.3 kV respectively. Mass spectrometer method was operated in positive ion mode set to go through 3626 cycles for 90 minutes, where each cycle performing one TOF-MS scan type (0.25 sec accumulation time, in a 350 to 1250 m/z window) followed by thirty information dependent acquisition (IDA)-mode MS/MS-scans on the most intense candidate ions having a minimum 150 counts. Each product ion scan was operated under vender specified high-sensitivity mode with an accumulation time of 0.06 secs and a mass tolerance of 50 mDa. Former MS/MS-analyzed candidate ions were excluded for 13 sec after its first occurrence, and data were recorded using Analyst-TF (v.1.5.1) software.

A merged search was performed from the combined 20 nLC-MS/MS analyses from the SCX fractions, using ProteinPilot software (version 4.2, revision 1297) that utilizes Paragon algorithm (ver 4.2.0.0, 1296) against UniProt/SwissProt database of *Mus Musculus* protein sequences (captured 6/22/10) supplemented with common contaminating proteins (human keratins, porcine trypsin, etc.) for a total of 48,054 proteins searched. ProteinPilot search parameters included all biological modification as variable modifications and carboxyamidomethyl cysteine was used as a fixed modification. Trypsin was used as the cleavage specificity with up to 2 missed cleavages. The precursor mass tolerance was automatically set by the ProteinPilot software based on the instrument type used—in this case a quadrupole-Tof instrument. The False Discovery Rate (FDR) was determined by the ProteinPilot/Paragon software to be less than 1% from a search of a decoy, inverse sequence database of the UniProt/SwissProt mouse database described above. Data normalization across all iTRAQ tag channels was accomplished in the ProteinPilot software using the bias correction function. This function evaluated the relative ratio of the iTRAQ tags across all the peptides identified and normalizes the ratios such that the collective ratio of all the tags is 1:1:1:1, prior to calculating relative differences among individual peptides/protein. For protein identification and quantitative profiling, a minimum 1.5-fold-change from a least 2 peptides at 99% or greater confidence was required. Raw data of proteomic analysis are presented in supplemental file (S1 File).

Differentially expressed proteins identified using either of the proteomics methods were analyzed through the use of QIAGEN’s Ingenuity Pathway Analysis suite (IPA, QIAGEN Redwood City, CA), as described elsewhere [18].

### Native gel electrophoresis and 2-dimensional BNGE

Mitochondria (0.5 mg protein) were solubilized in 50 μl of 140 mM NaCl, 2.7 mM KCl, 8 mM NaH_2_PO_4_, and 1.4 mM KH_2_PO_4_ (pH 7.4) in the presence of digitonin (4 mg digitonin per 1 mg mitochondrial protein for 30 min in ice. Solubilized mitochondria were centrifuged at 15,000xg for 30 min at 4°C. Supernatants were first subjected to BNGE (blue-native gel electrophoresis, first dimension) on NativePAGE Novex Bis-Tris gel system (Life Technologies). Strips of native gel containing proteins were cut, equilibrated with SDS-loading buffer, overlaid on top of NuPAGE Novex Bis-Tris 4–12% gradient gel with 2D well (Life Technologies), and the proteins were separated under denaturing conditions (second dimension). All procedures were performed as described by the manufacturer. Proteins were transferred to nitrocellulose membrane, and mitochondrial complexes were visualized using a cocktail of antibodies specific to mitochondrial ETC complexes (Life Technologies, Cat. # 457999), as described below.

### Western blot analysis

Western immunoblot analyses were done by standard techniques using NuPAGE Novex Bis-Tris pre-cast gels (Life Technologies). Protein samples were solubilized in 100 mM KH_2_PO_4_, 2% Triton X-100, containing a protease inhibitor cocktail (Roche) at a concentration of 2 μg protein/μl and cleared by centrifugation at 12000xg for 10 minutes at 4°C. One volume of 4X SDS-PAGE loading buffer (Life Technologies) was added to 3 volumes of samples. Electrophoresis was performed for 2 h at 125 V. Proteins were transferred to nitrocellulose membranes using the semi-dry transfer method.

Membranes were blocked overnight at 4°C with agitation using 1x TBS (10 mM Tris, 150 mM NaCl) supplemented with 5% BSA. Membranes were then washed 3x5 min with 0.02% Tween-20 in TBS and incubated for 1 hour at room temperature with primary antibodies. ETC bands were detected with a commercial monoclonal antibody cocktail specific to components of ETC complexes (Life Sciences, Cat # 457999, dilution 1:1000). These antibodies detect 70 kDa SDHB flavoprotein of C-II, 55 kDa ATP5A subunit of C-V, 48 kDa UQCRC2 subunit of C-III, 39 kDa NDUFA9 subunit of C-I and 15 kDa COX4 subunit of C-IV. Commercial primary antibodies specific to myoglobin (Santa Cruz, sc-25607, dilution 1:1000), Rieske subunit (Abcam, ab14746, dilution 1:500), Mthfd2 (Pierce, PA5-28169, dilution 1:1000) and our homemade polyclonal antibodies against medium-chain acyl-CoA dehydrogenase (MCAD), very long-chain acyl-CoA dehydrogenase (VLCAD), long-chain 3-hydroxyacyl-CoA dehydrogenase (LCHAD), and mitochondrial malate dehydrogenase (mMDH) were used. Next, membranes were washed 3x with 0.02% Tween-20 in TBS. Secondary IRDye antibodies were used for imaging (Licor Biosciences, Cat. # 827–08364 and 926–68171). The secondary antibodies were used at a concentration of 1:20,000, with an incubation period of 1 h at room temperature with agitation. The final wash of 3x5 min with 0.02% Tween-20 in TBS culminated in a wash using imaging buffer composed of 1x TBS without Tween-20. The Odyssey CLx scanner was used for imaging of membranes in the 800 nm and 700 nm channels. Band intensity analysis and data quantification were done with Image Studio software (LiCor biosciences).

### Ultracentrifugation of mitochondria on discontinuous sucrose gradients

Mitochondria (3.5–4 mg protein) were solubilized in 500 μl of 140 mM NaCl, 2.7 mM KCl, 8 mM NaH_2_PO_4_, 1.4 mM KH_2_PO_4_ (pH 7.4) in the presence of 1% lauryl-maltoside for 30 min in ice [19, 20]. Solubilized mitochondria were centrifuged at 15,000xg for 30 min at 4°C. Approximately 450 μl of supernatant was loaded on a 10–35% discontinuous sucrose gradient in the presence of 0.05% lauryl maltoside. Samples were subjected to centrifugation at 175,000xg for 20 h at 4°C. Gradient fractions were collected and snap frozen in liquid nitrogen and kept at -80C for further Western blot analysis.

### Immunohistochemistry

Animals were euthanized by an overdose of isoflurane and IV injection of cardioplegic solution composed of 68 mM NaCl, 50 mM KCl, 12 mM NaHCO_3_, 11 mM glucose, 30 mM 2,3-butanedione monoxime, 10 mM EGTA, 10 U/L heparin, and 0.2 mg/L nifedipine. Hearts were removed, briefly perfused with cardioplegic solution and embedded in the OCT medium. Cryosections (5 um) were postfixed with cold acetone for 10 minutes. Sections were blocked for 1 h in a blocking reagent composed of 3% normal goat serum, 2% BSA, 0.15% Triton X-100 in PBS. Slides were incubated overnight with polyclonal anti-MTHFD2 antibodies (Pierce, PA5-28169) and monoclonal antibodies specific to mitochondrial complex-I subunit NDUFA9 (Abcam, Ab14713), followed with a mixture of secondary goat-anti-rabbit IgG Alexa Fluor 488 antibodies and goat-anti-mouse IgG Alexa Fluor 594 (Life Technologies, A11006 and A11005, respectively). Nuclei were visualized with DAPI (Vector Laboratories). Slides were imaged using a Nikon A1-R confocal microscope with NIS-Elements software (Nikon, Japan).

### Quantitative RT-PCR

Total mRNA was extracted from cardiac muscle using TRI reagent (Sigma, T9424) and reverse transcribed into cDNA by using a Script cDNA Synthesis Kit (Bio-Rad, 170–8891). RT-PCR was performed using a Realplex Mastercycler (Eppendorf) and SYBR Green RT-PCR reagent (Bio-Rad 172–5121). PCR conditions were as follows: 30 s at 95°C, 30 s at 55°C, and 40 s at 68°C for 35 cycles. After amplification, a melting curve (0.01 C/s) was used to confirm product purity. Ribosomal protein 7 mRNA (RLP7) was used as an internal control. Results are expressed relative to RLP7. The oligonucleotide primers used to carry out the PCRs are described in the Table 1.

**Table 1:** Oligonucleotides used for quantitative PCR analyses.

Gene	Forward	Reverse
Mouse Mthfd2	5’-AAGAATGTGGTAGTGGCTGG-3’	5’-AAGAATGTGGTAGTGGCTGG-3’
Mouse Taz	5’-ATGCCCCTCCATGTGAAGTG-3’	5’-TGGTTGGAGACGGTGATAAGG-3’
Mouse RLP7	5’-GAAGCTCATCTATGAGAAGGC-3’	5’-AAGACGAAGGAGCTGCAGAAC-3’

### Statistical analysis

Differences between groups were assessed for significance by the unpaired Student’s *t*—test with the assumption of equal variances. The results were considered statistically significant if the P value was ≤ 0.05. Results are expressed as arithmetic means ± SEM.

## Results

### Mitochondrial proteomic alterations reveal metabolic remodeling in the TazKD mouse heart

To investigate molecular events in mitochondria at the proteomic level that precede the apparent cardiomyopathic phenotype, ventricular myocardium of 3-month-old TazKD male mice was used. To minimize the impact of possible side effects of doxycycline on the mitochondrial proteome, both control and TazKD mice were maintained on the doxycycline-containing rodent chaw (625 mg dox/kg chaw).

**Fig 2:**
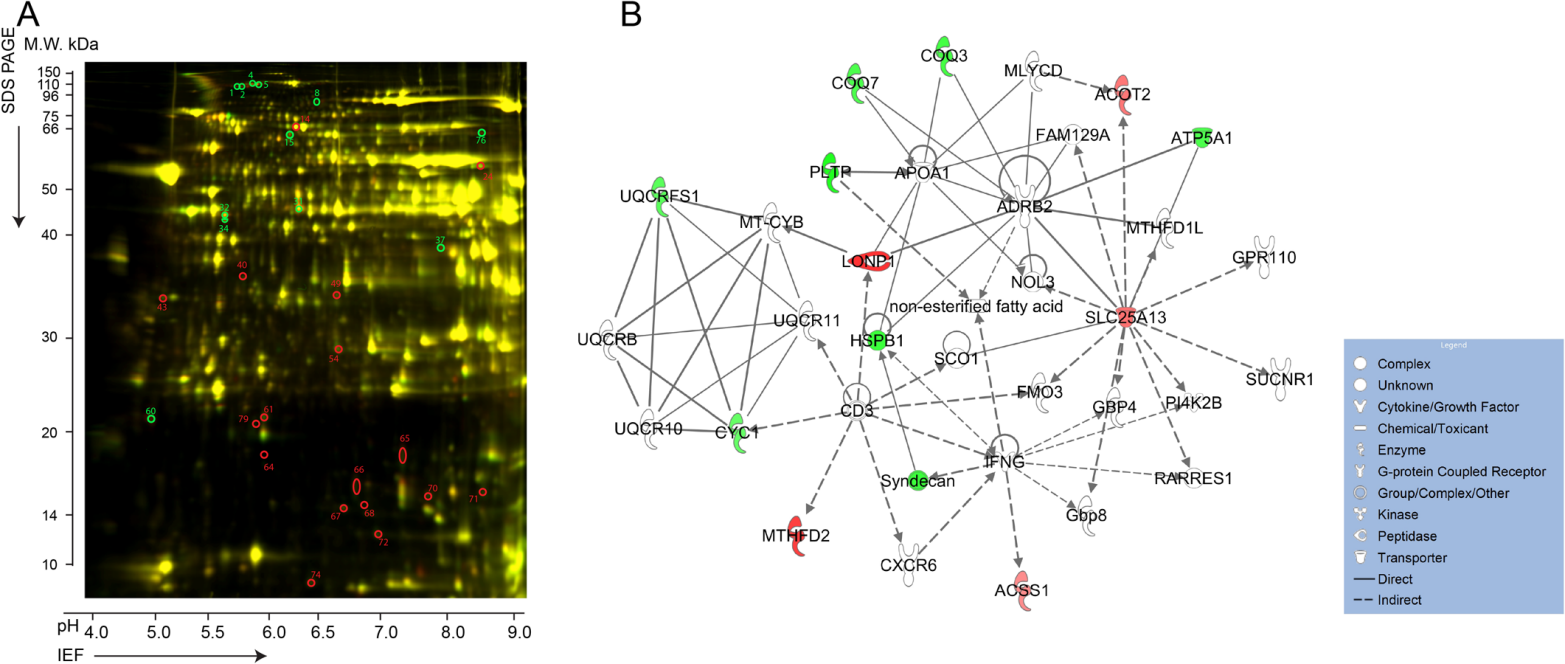
(A) Representative 2D-DIGE image of differentially labeled proteins in mitochondria from 3-month-old wild type and TazKD mice. Proteins with changes in expression levels of at least 1.5-fold are marked with red (decreased) and green (increased) circles. Spot numbers correspond to those in Table 1. (B) Top-rated network of proteins involved in lipid metabolism and associated with altered mitochondrial proteins in TazKD hearts. Shapes denote functional classes of proteins. Shapes in green depict down-regulated proteins; red-colored symbols indicate up-regulated proteins. Proteins in white are theoretically identified by Ingenuity Pathway Knowledge Base analysis. Solid and dashed lines depict direct and indirect interactions, respectively.

Mitochondria were isolated from ventricular myocardium of 2 WT and 2 TazKD mice and subjected to 2D-DIGE analysis. The DeCyder software detected more than 2100 matched spots on each 2D gel, among which the relative abundances of 82 spots were consistently altered between the control and TazKD groups (Fig 2A). From these 82, we selected 31 spots with ≥1.5 fold change (18 decreased and 13 increased, p ≤0.05) between TazKD and WT preparations and subjected them to MALDI-TOF/TOF MS analysis for protein fingerprinting analysis. A Mascot database search of MS spectra resulted in the identification of 24 non-redundant mitochondrial polypeptides.

iTRAQ analysis, as depicted in Fig 1, identified a total of 533 polypeptides, including 5 mitochondrial proteins that were differentially represented in the WT and TazKD samples with a greater than a 1.5-fold change (i.e. >33% decrease in signal or >50% increase in signal with respect to the WT with 99% confidence). The combined results of proteomic studies, including 17 decreased and 9 increased non-redundant proteins are presented in Table 2. All 26 proteins were related to specific metabolic pathways, including oxidative phosphorylation, ubiquinone biosynthesis and tetrahydrofolate-coupled one-carbon metabolism (Table 2). Enzymes involved in oxidative phosphorylation and ubiqunone biosynthesis pathways were most notably reduced in TazKD mitochondria. Subunits of cytochrome *bc1* complex (UQCRFS1 and CYC1), chaperone involved in cytochrome *bc1* assembly (ISCU) and kinase involved in ubiquinone synthesis (ADCK3) were most significantly downregulated in TazKD samples.

**Table 2:** Differentially expressed mitochondrial proteins identified by 2D-DIGE and iTRAQ analyses TazKD mice.

Spot number	Entrez Gene Name	Gene	UniProt	C.I. %	Fold change	Metabolic pathways
**Decreased in TazKD**						
64	Phospholipid transfer protein	*Pltp*	P55065	98	2.19	Lipid transport, LXR/RXR activation
51	Heat shock protein beta-1	*Hspb1*	P14602	100	1.91	Cellular stress response
40	Hexaprenyldihydroxybenzoate methyltransferase	*Coq3*	Q8BMS4	97	1.83	Ubiquinone biosynthesis
71	Cyclophilin D	*Ppif*	Q99KR7	100	1.65	Apoptosis, PTP-regulation
61	Succinyl-CoA:3-ketoacid-coenzyme A transferase 1	*Oxct1*	Q9D0K2	100	1.79	Metabolism of ketone bodies
79	Ubiquinone biosynthesis protein COQ7 homolog	*Coq7*	P97478	100	1.74	Ubiquinone biosynthesis
24	ATP synthase subunit alpha	*Atp5a1*	Q03265	100	1.75	Oxidative phosphorylation
72	Iron-sulfur cluster assembly enzyme ISCU	*Iscu*	Q9D7P6	100	1.70	Oxidative phosphorylation
43	Ubiquinone biosynthesis protein COQ9	*Coq9*	Q9D7P6	100	1.62	Ubiquinone biosynthesis
65	Cytochrome b-c1 complex subunit Rieske	*Uqcrfs1*	Q9CR68	100	1.60	Oxidative phosphorylation
54	Peroxiredoxin-5	*Prdx5*	P99029	100	1.72	Cellular response to oxidative stress
70	Succinate dehydrogenase flavoprotein	*Sdha*	Q8K2B3	100	1.59	Oxidative phosphorylation
14	NADH dehydrogenase subunit 5	*Ndufa5*	Q9CPP6	100	1.54	Oxidative phosphorylation
74	Cytochrome c1, heme protein	*Cyc1*	Q9D0M3	100	1.50	Oxidative phosphorylation
49	Methylmalonyl-CoA epimerase	*Mcee*	Q9D1I5	100	1.64	Metabolism of branched amino acids
67	Myoglobin	*Mb*	P04247	100	2.03	Intracellular oxygen transport
66	Myoglobin	*Mb*	P04247	100	1.82	Intracellular oxygen transport
68	Myoglobin	*Mb*	P04247	100	1.44	Intracellular oxygen transport
i-113	Chaperone activity of bc1 complex-like	*Adck3*	Q60936	>99	1.62	Ubiquinone biosynthesis
**Increased in TazKD**						
8	Delta-1-pyrroline-5-carboxylate synthase	*Aldh18a1*	Q9Z110	100	2.11	Biosynthesis of proline, ornithine and arginine
15	Delta-1-pyrroline-5-carboxylate synthase	*Aldh18a1*	Q9Z110	100	1.85	Biosynthesis of proline, ornithine and arginine
32	Mitochondrial inner membrane protein (mitofilin)	*Immt*	Q8CAQ8	100	1.63	MICOS complex subunit, inner membrane architecture, crista junctions
34	Succinyl-CoA ligase subunit beta	*Suclg2*	Q9Z2I8	100	1.79	TCA cycle, carbohydrate metabolism
31	Succinyl-CoA ligase subunit beta	*Suclg2*	Q9Z2I8	100	1.97	TCA cycle, carbohydrate metabolism
4	Acyl-coenzyme A thioesterase 2	*Acot2*(*)	Q9QYR9	100	1.96	Acyl-CoA metabolism
i-70	Acyl-coenzyme A thioesterase 2	*Acot2* (*)	Q9QYR9	>99	3.29	Acyl-CoA metabolism
1	Lon protease homolog	*Lonp1*(*)	Q8CGK3	100	2.95	Degradation of misfolded, polypeptides. Chaperone activity
2	Lon protease homolog	*Lonp1*(*)	Q8CGK3	100	2.41	Degradation of misfolded, polypeptides. Chaperone activity
i-21	Lon protease homolog	*Lonp1*(*)	Q8CGK3	>99	2.77	Degradation of misfolded, polypeptides. Chaperone activity
37	Mitochondrial 10-formyltetrahydrofolate dehydrogenase	*Aldh1l2*	Q8K009	100	2.31	Tetrahydrofolate / One carbon metabolism
76	Mitochondrial 10-formyltetrahydrofolate dehydrogenase	*Aldh1l2*	Q8K009	100	2.60	Tetrahydrofolate / One carbon metabolism
5	Bifunctional methylenetetrahydrofolate dehydrogenase	*Mthfd2*(*)	P18155	100	2.76	Tetrahydrofolate / One carbon metabolism
i-360	Bifunctional methylenetetrahydrofolate dehydrogenase	*Mthfd2*(*)	P18155	>99	2.86	Tetrahydrofolate / One carbon metabolism
60	Ca^2+^-binding mitochondrial carrier Aralar2	*Slc25a13*	Q9QXX4	100	2.06	Mitochondrial glutamate / aspartate transport
i-51	Acetyl-coenzyme A synthetase 2	*Acss1*	Q99NB1	>99	1.73	Acetyl-CoA biosynthesis, TCA cycle

(i-number)—Protein number from iTRAQ studies

(*)—Identified both in 2DIGE and iTRAQ studies.

Changes of proteomic landscape in L_4_CL-deficient mitochondria had most significant impact on C-III, reducing electron flow rate between C-I to C-III by 60%, while had little or no effect on activities of other ETC complexes [12, 14].

Bioinformatics analysis using IPA tools showed association of most altered proteins with mitochondrial dysfunction and lipid metabolism disorders and were clearly clustered with beta-2-adrenergic receptor (ADRB2), citrin (SLC25A13), and apolipoprotein A1 (APOA1) (Fig 2B). Defects in these genes are associated with various clinical conditions including obesity, type-2 diabetes (ADRB2), citrullinemia (SLC25A13), Tangier disease and systemic amyloidosis (APOA1) [21–24].

Increases in the relative abundances of enzymes such as MTHFD2, ALDH1L2, SLC25A13, LONP1, ALD18A1 and SUCB2 (Table 2) represent robust changes in proteins involved in metabolism of folates, amino acids, lipids and carbohydrates in L_4_CL-depleted mitochondria of TazKD hearts, suggesting an activation of anaplerotic reactions in cardiac energy producing systems such as protein and amino acid catabolism.

We observed marked increases of mitofilin (IMMT) and Lon protease (LONP) in TazKD samples (Table 2). Mitofilin is a part of the large inner mitochoncrial membrane (IMM) organizing system (MINOS). The MINOS complex is required for connecting cristae membranes to the inner boundary membrane and may be a central part of the even larger endoplasmic reticulum-mitochondria organizing network (ERMIONE), which also includes OPA1 [25]. Additionally, an outer membrane-bound mitofilin participates in protein import via the mitochondrial intermembranous space assembly pathway [26]. Mitochondrial ATP-dependent Lon protease, LONP1, mediates the selective degradation of misfolded, unassembled or oxidatively damaged polypeptides in the mitochondrial matrix. It may also have a chaperone function in the assembly of IMM protein complexes and participate in the regulation of mitochondrial gene expression and maintenance of the integrity of the mitochondrial DNA [27, 28].

### Perturbations in oxidative phosphorylation, assembly of respiratory chain complexes and the ubiquinone biosynthesis pathway

**Fig 3:**
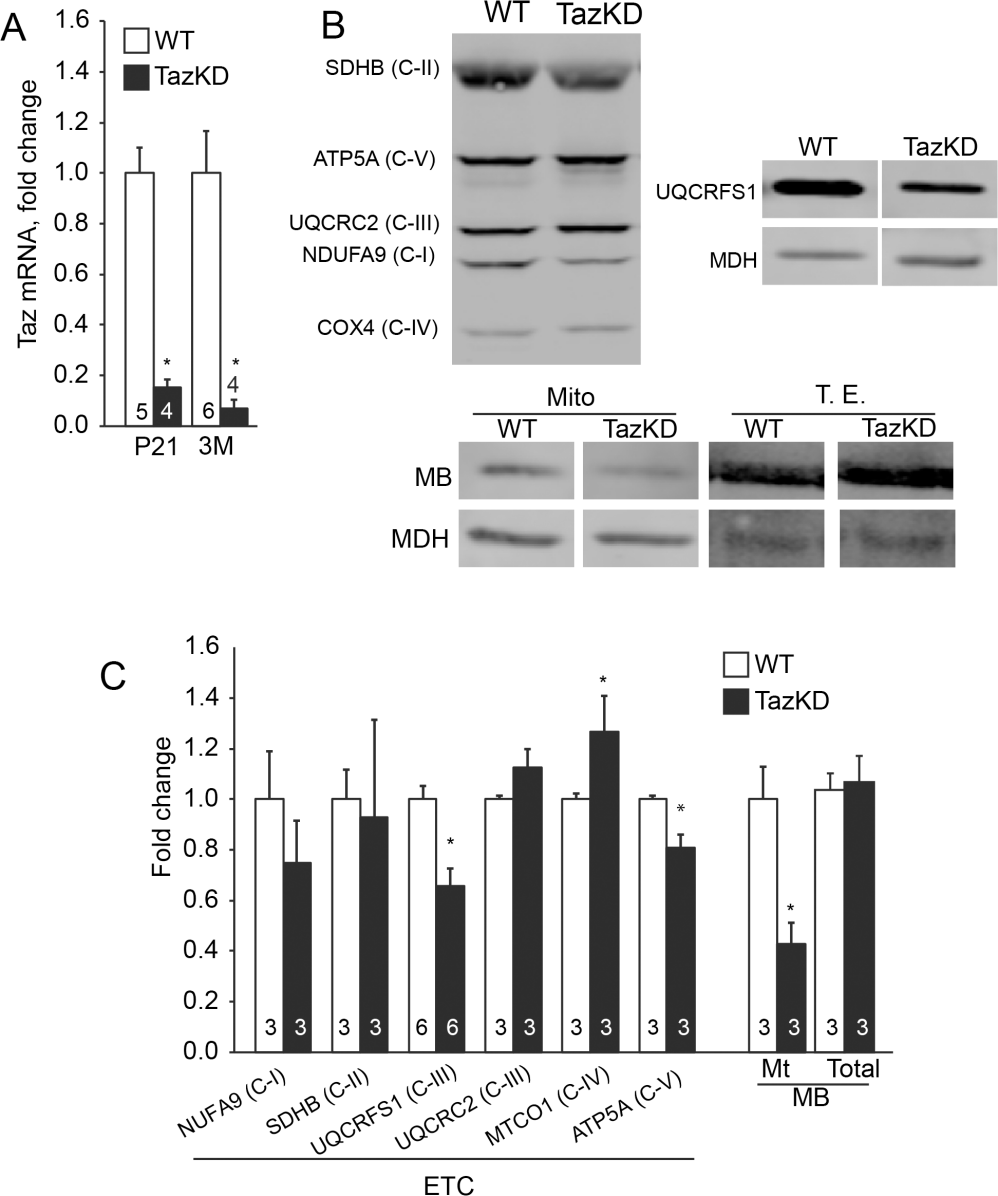
Quantitative analysis of mitochondrial ETC enzymes, myoglobin (MB) and MTHFD2 in cardiac mitochondria of TazKD mice. (A) Quantification of Taz mRNA levels in hearts of 3 weeks old (P21) and 3 months old (3M) WT and TazKD mice. The expression levels were determined by quantitative RT-PCR and represented as relative normalized expression. (B) Representative Western blots of mitochondrial proteins from TazKD and WT hearts. Mitochondrial proteins (30 μg) were separated by SDS-electrophoresis and subjected to Western blot analysis using a cocktail of monoclonal antibodies specific to individual polypeptides of ETC complexes NDUFA9 (C-I), SDHB (C-II), UQCRC (C-III), COX4 (C-IV) and ATP5A (C-V). Additionally, blots were probed with antibodies specific to Rieske Fe-S cluster protein (UQCRFS1) and myoglobin (MB). Myoglobin content was analyzed in mitochondrial fraction (Mito) and the total cardiac tissue protein extract (T.E.) fractions. Antibodies specific to mitochondrial malate dehydrogenase (MDH) were used as a loading control. The entire (uncropped) images of Western blots are shown in S1 Fig. (C) Quantitative assessments of Western blot analyses. Bars represent fold-changes in band intensities in TazKD samples compared to WT controls. For **A** and **C:** Data shown are means ± standard error of mean (SEM). Number of experiments per group is denoted in the corresponding bar. Asterisks indicate significant differences of P≤0.05 (Student’s t-test) between WT and TazKD.

We found nine polypeptides involved in oxidative phosphorylation, assembly of ETC complexes, and the ubiquinone biosynthesis pathway that were significantly decreased in TazKD hearts (Table 2). Among the ETC-forming polypeptides, most significantly reduced in L_4_CL-depleted mitochondria were the NDUFA5 subunit of complex I (C-I), the catalytic SDHA subunit of complex II (C-II), Rieske (UQCRFS1) and cytochrome *c1* (CYC1) subunits of complex III (C-III), and ATP5A1, the catalytic core-forming subunit ATPA of complex V (C-V) (Fig 3). Relative amounts of ancillary proteins involved in ubiquinone biosynthesis (COQ3, COQ7, COQ9, ADCK3) and Fe-S cluster assembly (ISCU) were also significantly reduced in L_4_CL-deficient mitochondria from TazKD hearts. Quantitative reduction of Fe-S cluster containing Rieske protein was confirmed with Western blot analysis (Fig 3B), and this was consistent with the previously reported diminished C-III activity in L_4_CL-deficient cardiac mitochondria from TazKD mice [12, 14].

Interestingly, mitochondria-associated myoglobin was markedly reduced in TazKD hearts (Fig 3B and 3C), while the total content of myoglobin in heart tissue was not altered. This suggests a possible blockade of intracellular oxygen transport from the plasma membrane to mitochondria in TazKD cardiomyocytes due to reduction of mitochondria bound myoglobin.

### ETC supercomplexes (SCs) perturbed in CL-deficient mitochondria

**Fig 4:**
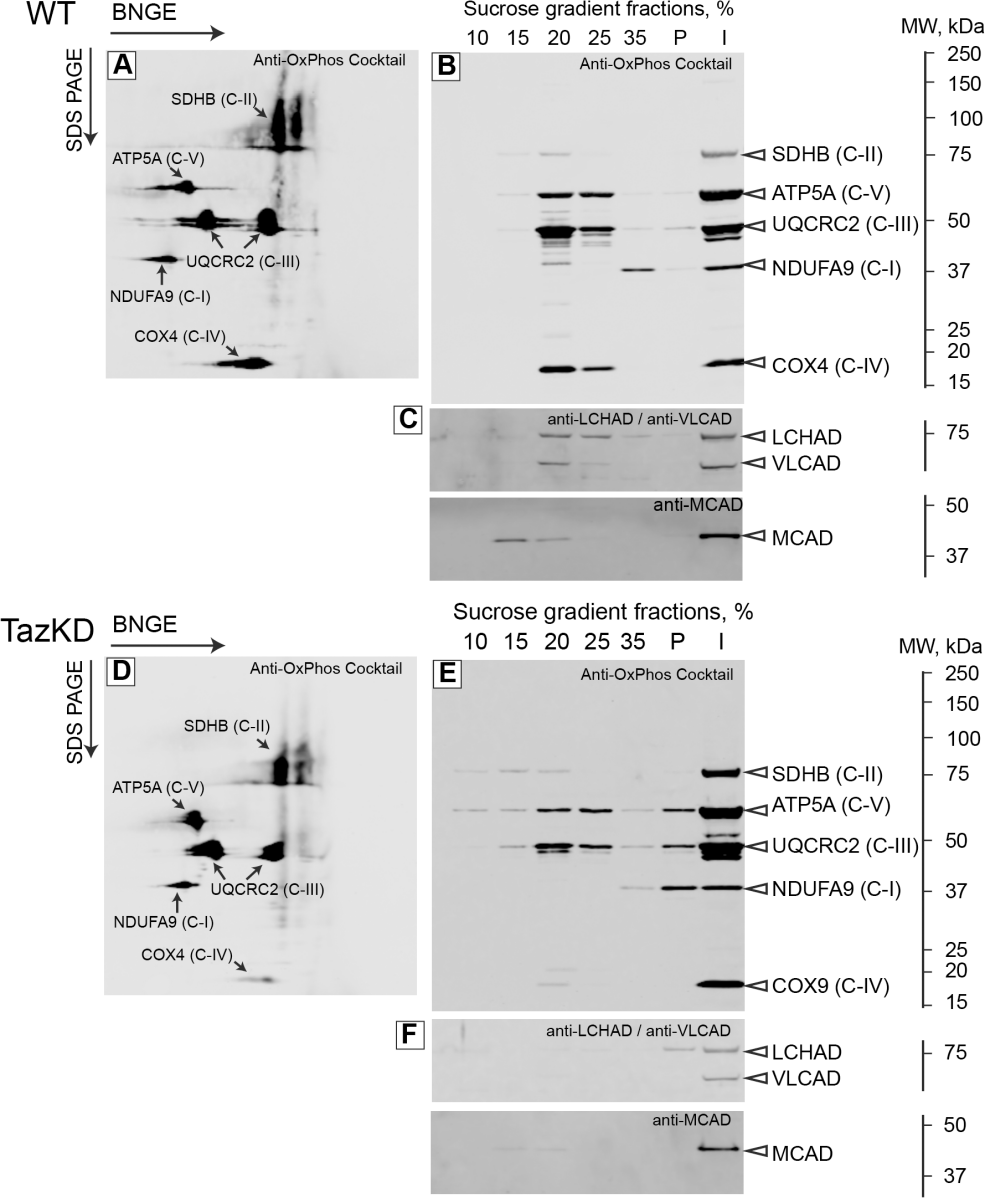
Evidence of destabilization of mitochondrial supercomplexes in cardiolipin-depleted cardiac mitochondria from TazKD mice. (A and D) Mitochondria from WT and TazKD hearts were solubilized with digitonin and subjected to blue-native gel electrophoresis (BNGE, first dimension) and then to SDS-gel electrophoresis under denaturing conditions (SDS-PAGE, second dimension). Gels were subjected to western blot analyses with an antibody cocktail specific to subunits of mitochondrial ETC complexes (Anti-OxPhos Cocktail). Bands representing the individual polypeptides that correspond to ETC complexes are marked. In a separate set of experiments, mitochondria from WT (B and C) and TazKD (E and F) hearts were solubilized with lauryl maltoside and subjected to sucrose gradient ultracentrifugation. Individual fractions of the gradient were subjected to western blot analyses using Anti-OxPhos Cocktail (B and E) or FAO enzyme-specific antibodies LCHAD, VLCAD and MCAD (C and F). P—pellet; I—input material (i.e. the solubilized mitochondria preparation that was applied to the sucrose gradient). The scales on the right side depict molecular masses in kilodaltons (kDa).

To study the effects of L_4_CL-deficiency on ETC supercomplex (SC) assembly, isolated mitochondria were gently solubilized with lauryl maltoside and centrifuged at 12,000xg at 4°C for 15 min, as described elsewhere [19, 20]. Supernatants containing SCs were collected and subjected to 2-dimensional blue native gel electrophoresis (2D-BNGE). First, mitochondrial complexes were separated by BNGE (first dimension) with subsequent SDS-PAGE under denaturing conditions (second dimension). Proteins were transferred onto nitrocellulose membranes and probed with a cocktail of monoclonal antibodies that are specific to individual components of ETC complexes. Position and intensities of bands of NDUFA9 (C-I), SDHB (C-II), UQCRC2 (C-III) and ATP5A (C-V) did not differ between WT and TazKD samples, as shown in Fig 4A and 4B. In contrast, samples of TazKD mitochondria contained a significantly reduced amount of COX4 subunit of C-IV, consistent with the previous report of reduced C-IV levels in lymphoblasts of BTHS patient [9].

To gain more detailed information on the role of L_4_CL on stability of ETC supercomplexes (SC), SCs were separated from gently solubilized mitochondria by ultracentrifugation on a discontinuous sucrose gradient. Individual sucrose gradient fractions were subjected to Western blot analysis with a cocktail of monoclonal antibodies specific to mitochondrial ETC complexes (Fig 4C and 4D). In wild type (WT) preparations, the majority of ETC complexes including C-III, C-IV and C-V and trace amounts of C-II, were present in the 20–25% sucrose fractions (Fig 4C). A substantial amount of NDUFA9 subunit of complex I was present in the 35% sucrose fraction, whereas the pellet fraction (P) contained only small amounts of NDUFA9, UQCRC2 and ATP5A corresponding to C-I, C-III and C-V, respectively. Distribution of ETC complexes between 20–35% sucrose fractions indicates that these complexes are associated with high-molecular mass structures in normal mitochondria. In contrast, only UQCRC2 subunit of C-III and ATP5A subunit of C-V were abundant in the 20–25% fractions in TazKD mitochondria. Strikingly, subunits of C-I and C-IV were almost absent in any gradient fractions, except a trace amount in the 35% fraction (Fig 4D). Interestingly, the insoluble pellet from TazKD samples contained substantial amounts of polypeptides of C-I, C-III and C-V compared to WT. Redistribution of ETC complexes into the pellet suggests that CL-deficiency causes accumulation of mitochondrial proteins into large insoluble or mis-folded structures, likely reducing or abolishing their enzymatic function.

### Interactions of supercomplexes with fatty acid oxidation (FAO) enzymes are disrupted in TazKD hearts

Recently, it has been demonstrated that complex I in liver mitochondria physically interacts with VLCAD, which catalyzes the first step of FAO, the major energy producing pathway in adult cardiomyocytes [29]. Another FAO enzyme, ACAD9, “moonlights” as a chaperone involved in the assembly of C-I [30] and ACAD9 mutations cause human C-I deficiency. LCHAD, encoded by the mitochondrial trifunctional protein alpha subunit gene, exhibits acyltransferase activity [31]. These findings imply the existence of very large metabolic units or “hypercomplexes” in mitochondria, consisting of FAO enzymes and ETC complexes, where FAO and oxidative phosphorylation pathways converge. Because the presence of CL in the IMM is essential for stabilizing ETC supercomplexes, it is apparently crucial for the stability of FAO-ETC interactions. Therefore, we investigated whether CL-deficiency affects interaction of ETC complexes with FAO enzymes. Western blot membranes with samples from individual sucrose gradient steps were probed with antibodies specific for the FAO enzymes, VLCAD, LCHAD and MCAD. VLCAD and LCHAD were enriched in the 20–25% sucrose fractions, along with C-I, C-II and C-IV, suggesting that approximately 45–50% of these enzymes are physically associated with high-molecular mass SCs (Fig 4E). In contrast, interaction of MCAD with SCs is minimal because less than 10% of the total loaded MCAD was in the 15% fraction and only a trace amount in the 20% fraction. The remaining MCAD was diluted across the entire 15.5 ml of sucrose gradient and was not detectable by Western blot analysis (Fig 4G). In TazKD mitochondria, LCHAD, VLCAD and MCAD were completely absent from any soluble fractions, and only a small amount LCHAD was present in a pellet (Fig 4F–4H). The absence of FAO enzymes in sucrose gradient fractions from CL-depleted mitochondria indicates that interactions between FAO enzymes and ETC SCs are abolished, suggesting the importance of CL in the interactions of FAO enzymes with ETC complexes in cardiac mitochondria.

### Activation mitochondrial folate / one carbon metabolism genes in TazKD hearts

**Fig 5:**
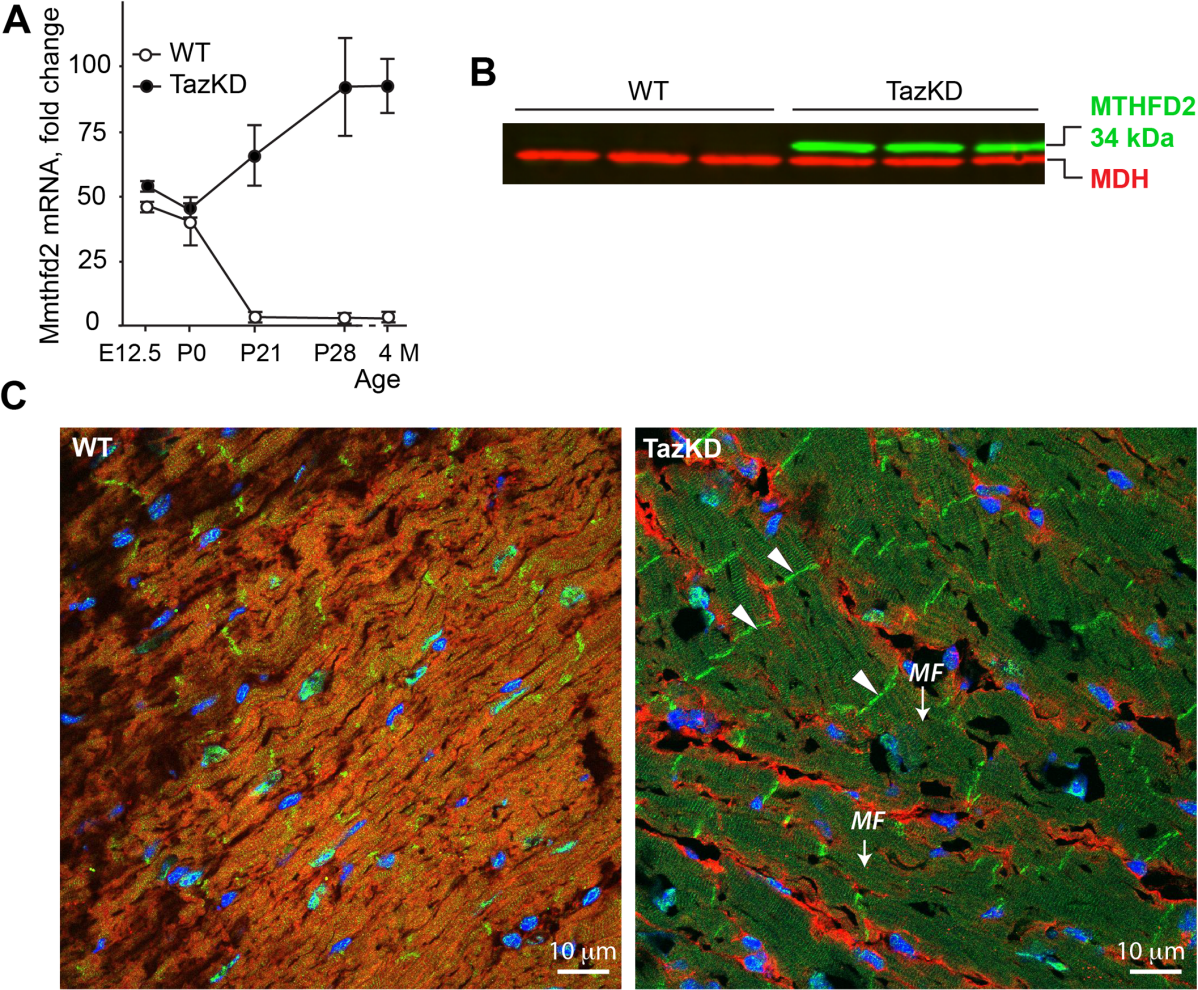
Altered Mthfd2 expression in adult TazKD heart. (A) Quantification of Mthfd2 mRNA levels in hearts of WT and TazKD mice during development. (B) Immunoblots of total protein extracts from 3 month-old WT and TazKD hearts with anti-MTHFD2 antibodies (green). Antibodies specific to mitochondrial malate dehydrogenase (MDH) were used as a loading control (red). (C) Immunofluorescent staining of MTHFD2 in LV sections of 3 month-old WT and TazKD mice revealing localization of MTHFD2 protein (green) in intercalated disks (arrowheads) and myofilaments (*MF*). Mitochondria were visualized with antibodies specific to complex I subunit NDUFA9 (red). Nuclei were stained with DAPI (blue).

We detected a robust increase in relative abundances of the MTHFD2 and ALDH1L2, enzymes that are involved in the folate metabolism. Folate is essential for the developing heart, providing one-carbon units for reactions that are necessary for synthesis of purine nucleotides and amino acids. Mthfd2 gene is abundantly expressed in fetal heart but after birth its expression quickly declines and becomes virtually undetectable by Western blot analysis in the adult hearts. In contrast, TazKD mice retain high level of MTHFD2 expression on both mRNA and protein levels (Fig 5A and 5B). In many cancer cells MTHFD2 is localized in mitochondrial [32], however its localization in adult cardiomyocytes of TazKD mice is unclear. Confocal immunofluorescent microscopy of cardiac muscle from adult mice corroborates results of our Western blot analysis: In WT hearts MTHFD2 is found only in few nuclei. In contrast, in TazKD hearts MTHFD2 is more abundant and is associated in myofilaments and at cellular junction sites, or intercalated disks (Fig 5C). Metabolic significance of MTHFD2 overexpression in adult TazKD hearts and its extramitochondrial localization at intercalated disks are unclear, but is likely to be a part of general activation fetal gene program in the pre-failing heart.

## Discussion

CL is essential for proper assembly and efficient functioning of ETC, oxidative phosphorylation, the protein import machinery, and mitochondrial carrier proteins [33–37]. TazKD mice present unique opportunities to elucidate changes in the mitochondrial proteomic landscape and metabolic pathways in the heart that are affected in Barth syndrome. We employed two complementary proteomic techniques, iTRAQ and 2D-DIGE, to analyze cardiac mitochondrial proteins in TazKD mice. All of the differentially expressed proteins identified in 2D-DIGE studies were also identified in iTRAQ experiments, except for ADCK3 and ACSS1. It is likely that the absence of TAZ among the altered proteins is because solubilization of TAZ requires extraction in high-pH buffer (unpublished).

### ETC complexes and CL-deficiency

**Fig 6:**
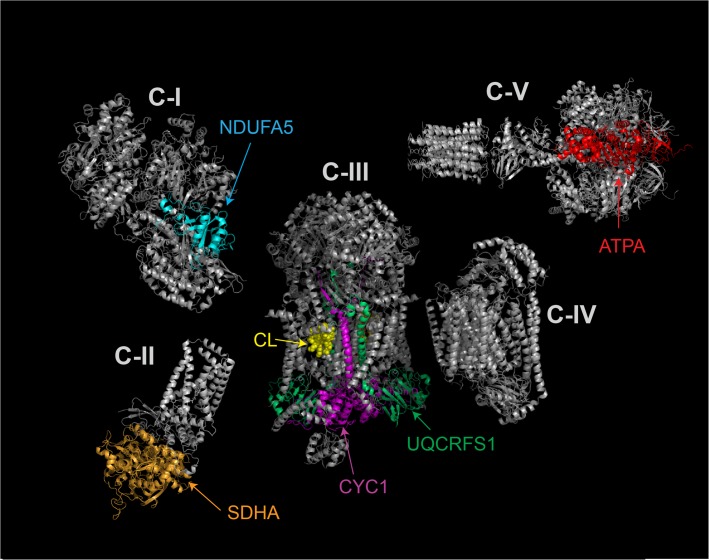
Structures of mitochondrial respiratory chain complexes that are affected in TazKD mice. Polypeptides that are quantitatively reduced in TazKD cardiac mitochondria are highlighted with colors. Structurally integrated cardiolipin (CL) molecules within complex III are highlighted in yellow.

Recently it has been demonstrated that the electron flow between C-I and C-III is reduced 2-fold, while enzymatic activity of CI is not affected, in CL-depleted mitochondria, suggesting that C-III is the most abnormal ETC complex in BTHS [12, 14]. The functional homodimer of mammalian C-III, (coenzyme Q:cytochrome c-oxidoreductase, also called the cytochrome *bc1* complex) is composed of 22 subunits which are encoded by both the mitochondrial (cytochrome *b*) and the nuclear genomes (all other subunits). We investigated which subunits of C-III are most significantly impacted by CL-deficiency. Proteomic studies with subsequent verification by Western blotting revealed that the relative abundance of UQCRFS1 (Fe-S cluster Rieske protein) was reduced 1.7 fold in BTHS mitochondria. Proteomic analysis also revealed a 1.7-fold reduction of ISCU (iron-sulfur cluster scaffold protein), which is involved in the biosynthesis of Rieske subunit and C-III assembly. The relative abundance of another C-III component, a heme containing cytochrome c1, was also significantly reduced in CL-deficient mitochondria. Mutations in the genes that encode components of C-III and ISCU protein are associated with myopathy and exercise intolerance, characteristic symptoms of human BTHS [38, 39]. Proteomic analysis also revealed that subunits NDUFA5 of C-I, SDHA of C-II and ATP5A1 of C-V were downregulated in CL-deficient mitochondria (Fig 6). We observed an approximately 35% (1.54-fold) reduction of peripheral 13 kDa subunit 5 of C-I that is encoded by *Ndufa5* gene. Downregulation of NDUFA5 protein has been reported in ischemic heart failure models [40]. NDUFA5 subunit is required for the formation of extramembrane arm of complex-I [41]. Knockout of *Ndufa5* gene in mouse neurons results only in partial, approximately 40% loss of C-I enzymatic activity [42]. Experimental findings showing that Taz knockdown has no effect on C-I enzymatic activity, as were reported in recent studies [12, 14], suggest that NDUFA5 is not directly involved in the catalytic process, but may be required for supercomplex assembly and stability. Same studies reported a negligible effect of Taz knockdown on mitochondrial ATP-synthase (C-V) activity, while our proteomic analysis revealed significant, almost 60% (1.75-fold) reduction of ATP5A1 in Taz-deficient mitochondria. The cause of these discrepancies is not clear. One possible explanation could be the contamination of mitochondrial samples by endothelial cell membrane fractions, where ATP synthase subunits are also present [43]. Overall, the fact that TazKD has negligible effects on the overall enzymatic activities of C-I, C-II, C-IV and C-V suggests that C-III is the principal target of CL-deficiency in BTHS mitochondria.

### Supercomplex assembly and stability

The mitochondrial respiratory chain, composed of individual ETC complexes I, III and IV in different stoichiometric ratios, is organized as large and dynamic supramolecular structures or supercomplexes [44]. Supercomplexes facilitate metabolic channeling of electron carriers (CoQ and cytochrome c) between individual complexes [45], and CL is essential for functional supercomplex formation in *S*. *cerevisiae* and mammalian mitochondria [33]. In bovine cardiac mitochondria, supercomplexes are composed of three individual complexes in the stoichiometric arrangement I_1_-III_2_-IV_1_ [44], while, in *S*. *cerevisiae*, the mitochondrial ETC lacks C-I, and a supercomplex is formed with C-III and C-IV mostly in the stoichiometric ratio III_2_-IV_1_. With limited direct protein-protein contacts and an average distance > 2 nm between complexes, it is most likely that supercomplexes are held together by protein-lipid rather than protein-protein interactions. It has been previously shown that each supercomplex could contain 200–400 molecules of CL [44], implying that CL is a scaffold for assembly of supercomplexes in mitochondria and essential for converging different metabolic fluxes. Supercomplexes are destabilized in CL-depleted mitochondria of lymphoblasts in BTHS patients. In particular, CL-deficiency causes dissociation of C-IV from supercomplex C-I/(C-III)_2_/C-IV, with concomitant accumulation of C-I/(C-III)_2_ in lymphoblasts from BTHS patients [9, 46].

Using a combination of blue-native electrophoresis with SDS-PAGE, we demonstrated that interactions between C-IV and complexes I-III, are destabilized in L_4_CL-depleted mitochondria. Further studies using ultracentrifugation of gently solubilized mitochondria on a sucrose gradient with subsequent Western blot analysis of gradient fractions confirmed the disassociation of C-IV from supercomplexes and revealed that interaction of C-I with C-III is also greatly compromised in L_4_CL-deficient mitochondria. Our proteomic studies revealed the reduction of COQ3 and COQ9 that are involved in ubiquinone biosynthesis. Reduction in these two enzymes would likely reduce the effective amount of ubiquinone available as an electron carrier between C-I and C-III and, therefore, would further diminish the efficiency of electron flow in mitochondrial ETC.

Considering the relatively modest changes in major ETC polypeptides, it appears that differences between WT and TazKD are more related to the assembly of the complexes and supercomplexes. Most of the major proteins are still present but they are not organized properly in TazKD mitochondria.

Together, diminished amounts of Rieske and cytochrome c1 subunits in C-III, in the setting of destabilization of ETC SCs, and the potentially reduced amount of available CoQ would be detrimental for efficient electron flux, generation of proton gradient across mitochondrial membrane, and, ultimately, for aerobic ATP synthesis.

### Fatty acid oxidation and CL-deficiency

Physical interaction of two long chain FAO enzymes, VLCAD and ACAD9 has been reported recently [29, 30]. It has been proposed that interaction of FAO enzymes with ETC complexes facilitates an efficient transfer of reducing equivalents from VLCAD via electron transfer flavoprotein (ETF) to ETF-dehydrogenase, resulting in reduction of CoQ (substrate of C-III). Additionally NADH, a product of the L-3-hydroxyacyl-CoA dehydrogenation reaction that is catalyzed by LCHAD in FAO, may also be channeled to C-I without a significant release into the mitochondrial matrix [29]. Co-migration of VLCAD and LCHAD in gradient fractions with C-I, C-III and C-IV suggests the physical interaction of long-chain FAO enzymes with a mitochondrial supercomplex. Disruption of FAO-ETC interactions in TazKD mitochondria suggests that CL is involved in the assembly of VLCAD-ETC and LCHAD-ETC supercomplexes, forming higher order metabolic units, or “hypercomplexes”. We previously demonstrated that WT mice are capable of dynamic switches of their metabolic fuel preferences from glucose to fatty acids when subjected to aerobic exercise, resulting in a sudden drop in respiratory exchange ratio values in indirect calorimetric measurements. In contrast, TazKD mice lack this adaptive mechanism, and carbohydrate metabolism continuously surges up in response to increased workload [14]. It is plausible that the physical interaction between FAO enzymes and ETC complexes is a pre-requisite for this metabolic switch.

### Myoglobin as an oxygen carrier and CL-deficiency

Our results reveal that mitochondrial-bound myoglobin is decreased more than 2-fold, while the total cellular myoglobin level remains unaffected in the hearts of TazKD mice. Myoglobin is the principal intracellular oxygen carrier that transfers oxygen molecules from the plasma membrane to mitochondria. Myoglobin is necessary to support cardiac function during development of zebrafish and mice, but adaptive cardiac remodeling can fully compensate for the deficit in cellular oxygen transport in myoglobin-knockout mice [47, 48]. Myoglobin does not have any known specific receptor on mitochondria, and its binding to the mitochondrial membrane is likely mediated by the electrostatic interaction of positively charged oxymyoglobin molecules with the negatively charged mitochondrial membrane.

It is feasible that oxymyoglobin interaction with CL-depleted mitochondria is reduced, diminishing the effective amount of available oxygen to the mitochondrial respiratory chain. While this hypothesis requires further study, our data on reduced mitochondrial-bound myoglobin are consistent with the recently reported results obtained from human subjects with BTHS. When stressed with aerobic exercise on the treadmill, participants with BTHS had markedly reduced ability to extract blood oxygen compared to unaffected participants, even though blood was oversaturated with oxyhemoglobin [49]. The reduced effective concentration of oxymyoglobin at CL-depleted mitochondrial membrane in combination with dissociation of C-IV from SCs may explain the reduced oxygen extraction capability in BTHS patients.

### Folate / one carbon metabolism and CL-deficiency

MTHFD2 is a rate-limiting enzyme of mitochondrial folate-coupled, one-carbon metabolism and is essential for developing embryos, but becomes silenced in adulthood, even in proliferative tissues [50, 51]. Silencing of the Mthfd2 gene after birth coincides with the loss of proliferation potential of neonatal mouse heart (Fig 5A). Folate and one carbon metabolism pathways are intimately coupled with methionine cycle and regulate homocysteine levels in tissues and plasma [52] [reviewed in [53]]. Perturbations in folate metabolism during prenatal development cause severe neural tube defects in humans and mice [50, 54, 55]. Interestingly, methyl donor deficiency in rats induces cardiomyopathy with imbalanced acetylation / methylation of PGC1α, decreased complex I activity and impaired mitochondrial fatty acid oxidation [56]. Activation of mitochondrial folate metabolism in TazKD harts is corroborated by recently published RNAseq-based gene expression analysis of TazKD hearts [12]. Activation of Mthfd2 gene expression in adult TazKD cardiomyocytes causes accumulation of MTHFD2 protein in myofilaments, which is consistent with its localization in the intermyofibrillar mitochondria. Surprisingly, MTHFD2 protein is also present in intercalated disks at intercellular junctions (Fig 5C). The biological significance of overexpression of Mthfd2 gene in Taz-deficient cardiomyocytes is unclear. However, robust activation of Mthfd2 gene has been detected in the skeletal muscles of knockout mice that are deficient in the mitochondrial helicase, Twinkle, and exhibit multiple mitochondrial DNA deletions and respiratory chain deficiencies [57], suggesting that activation of mitochondrial folate / one carbon metabolism may represent a general compensatory response to mitochondrial defects in striated muscles.

THF-1C metabolism, along with pentose phosphate cycle, is a major production route of NADPH, which is involved in ROS detoxification [58]. Mitochondrial folate mediated one carbon metabolism is coupled with oxidation of one carbon units derived from serine, glycine or sarcosin [see [32] for review]. Interestingly, Mthfd2 activation is accompanied with a quantitative increase of glutamate/aspartate carrier SLC25A13, perhaps echoing an increased mitophagy and protein / amino acid catabolism in TazKD cardiomyocytes [11]. Increased amino acid transport in TazKD mitochondria may also explain higher than WT respiration of TazKD mitochondria when glutamate and malate were used as substrates [12].

In present studies transgenic mice with tet-off system were used to achieve an efficient silencing of Taz gene. Mice received approximately 100 mg doxycycline x kg bw-1 x day-1. It is known that doxycycline is toxic for mitochondrial metabolism in liver, affecting oxidative phosphorylation rates and gene expression levels of key mitochondrial regulators including Tfam, sFis1, Mfn2, and Opa1 [59]. To minimize the possible side effects of doxycycline on the mitochondrial proteome, both control and TazKD mice were maintained on the doxycycline-containing rodent chaw. Nevertheless, we cannot completely exclude the effects of doxycycline-induced toxicity on cardiac mitochondria. It is possible that TazKD mice are more sensitive to doxycycline-induced cardiac injury than WT controls.

**Fig 7:**
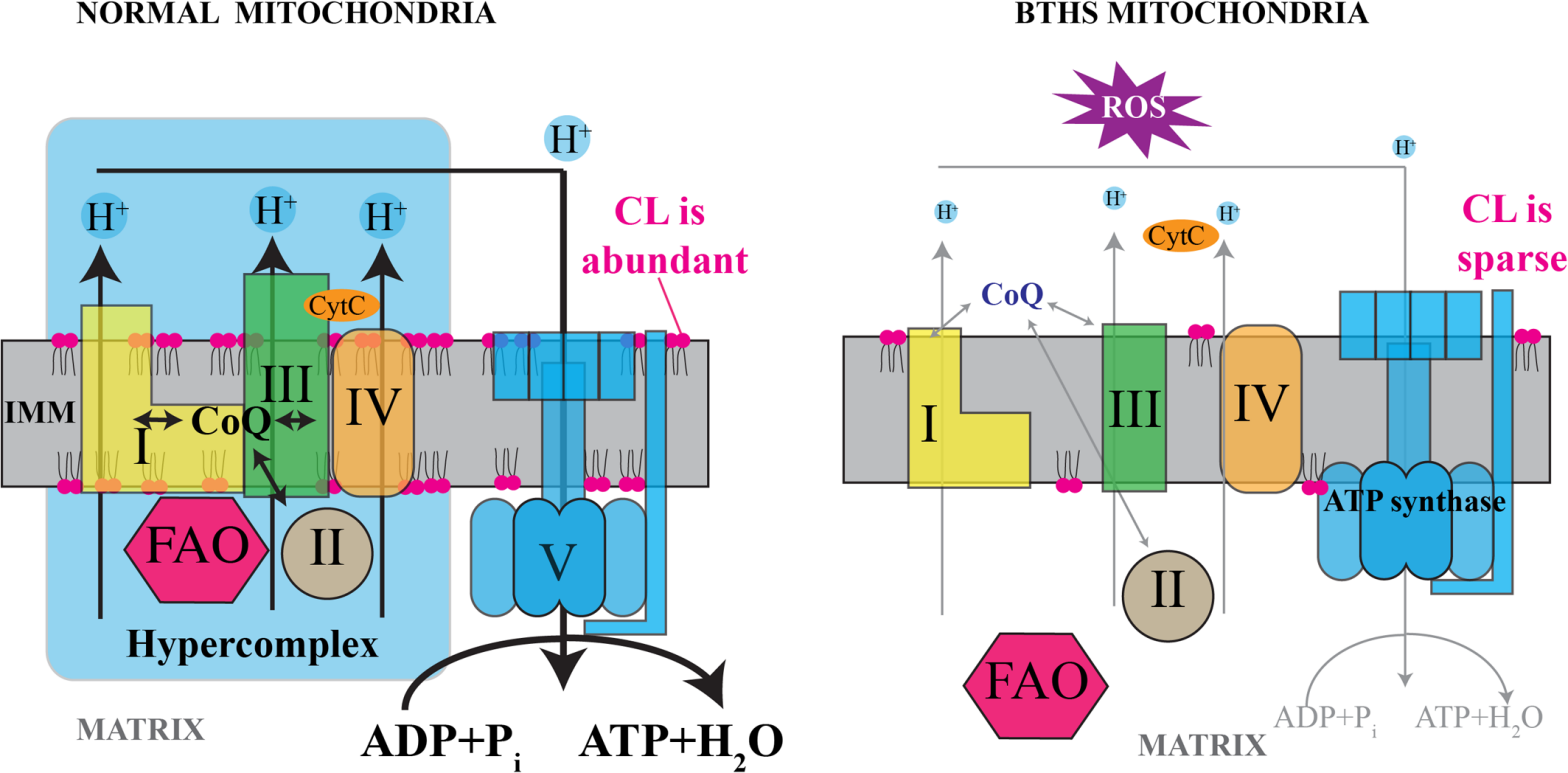
Hypothetical pathophysiological mechanism of Barth syndrome. In normal mitochondria with high content of CL in the IMM, respiratory complexes are assembled into supercomplexes and form even higher-order structures (hypercomplexes) by interacting with FAO enzymes. In contrast, in CL-depleted mitochondria from BTHS hearts, supercomplexes are destabilized, and the relative quantities of NDUFA5 (C-I), SDHA (C-II), UQCRFS1 and CYC1 (C-III) and ATPA (C-V) are reduced. Defects in respiratory chain complexes would likely induce overproduction of reactive oxygen species (ROS). In CL-deficient mitochondria, disassociation of FAO enzymes from RC complexes would reduce the efficiency of metabolic channeling through these pathways. Reduced oxygen availability at the outer mitochondrial membrane (OMM) due to low myoglobin binding to mitochondria would further diminish aerobic ATP synthesis, but might also reduce generation of ROS.

In summary, we have identified and quantitated the changes in cardiac proteome of mouse model of human Barth syndrome. Our findings indicate that Taz knockdown in mice diminishes mitochondrial ETC complex III activity, destabilizes ETC supercomplexes and disrupts interaction between ETC and FAO systems (Fig 7). The identification of a set of metabolic pathways affected by tafazzin deficiency in mitochondria, as well as adaptive changes to compensate for mitochondrial defects, represents a framework for the development of therapeutic strategies to ameliorate outcomes in this enigmatic disease.

## Supporting Information

S1 Fig Entire Western blots for cropped images shown in Fig 3B. (TIF)

S1 File Raw data of proteomic analysis. (XLSX)
