# Supplementary figures and images for: Cardiac Metabolic Pathways Affected in the Mouse Model of Barth Syndrome
Source: PLoS One. 2015 Jun 1;10(6):e0128561. doi: 10.1371/journal.pone.0128561 (PMC4451073; doi:10.1371/journal.pone.0128561)

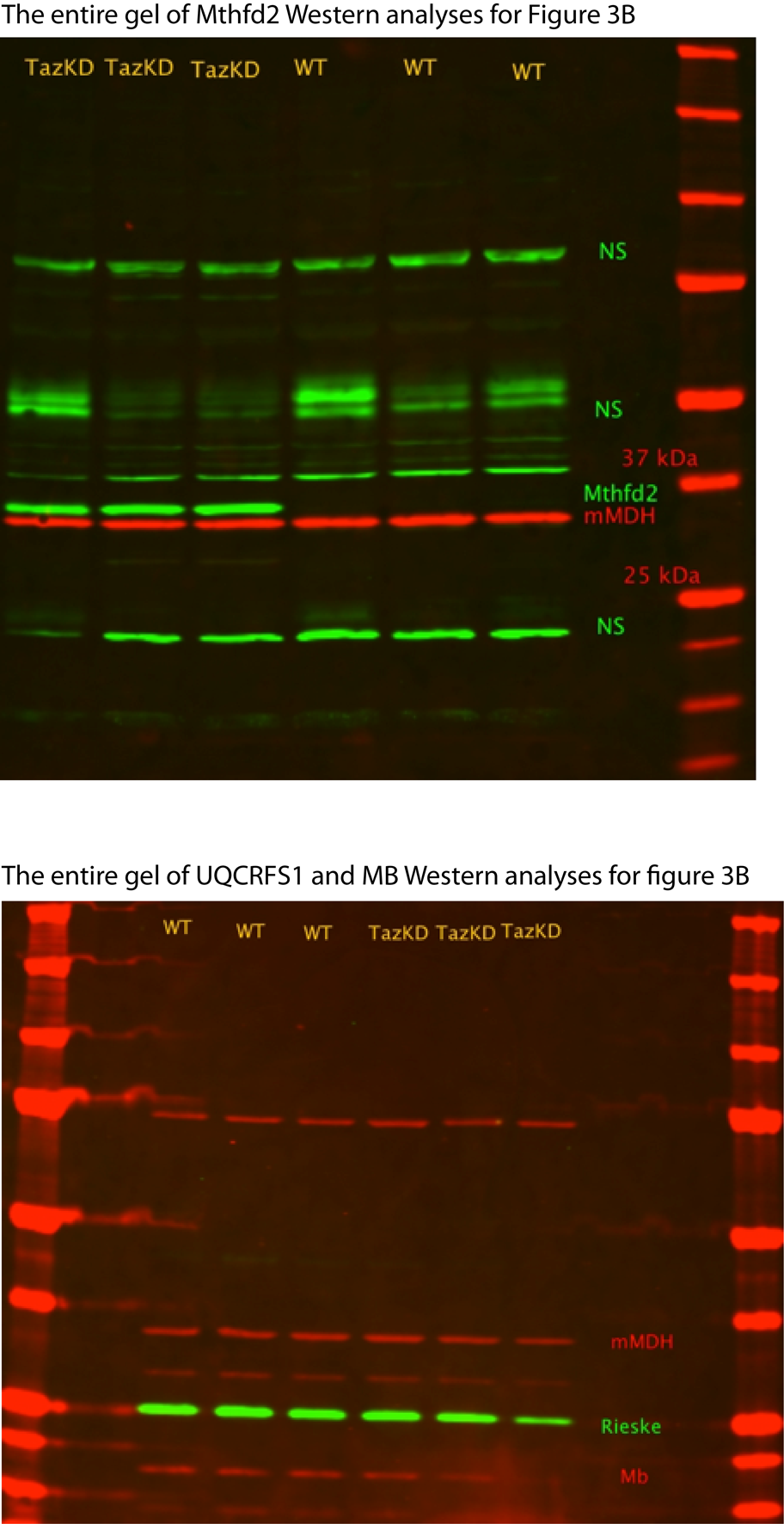

Supplement: S1 Fig — (TIF) [file pone.0128561.s001.tif]
